# DDX3X and virus interactions: functional diversity and antiviral strategies

**DOI:** 10.3389/fmicb.2025.1630068

**Published:** 2025-06-18

**Authors:** Shengming Ma, Qian Mao, Shaoting Weng, Man Teng, Jun Luo, Kunpeng Zhang

**Affiliations:** ^1^Henan Joint International Research Laboratory of Veterinary Biologics Research and Application, Anyang Institute of Technology, Anyang, China; ^2^Institute for Animal Health & UK-China Centre of Excellence for Research on Avian Diseases, Henan Academy of Agricultural Sciences, Zhengzhou, China

**Keywords:** DDX3X, virus, immune response, molecular interaction, cross-species heterogeneity

## Abstract

As a core member of the DEAD-box helicase family, DDX3X modulates RNA metabolic networks through its ATPase activity, RNA helicase function, and nucleic acid-binding capacity to participate in bidirectional regulation of innate immune responses and virus-host interactions. Multiple viruses achieve effective genome replication and immune evasion by hijacking DDX3X’s enzymatic activities or interfering with its mediated immune signaling transduction. Nevertheless, hosts have evolved strategies to exploit DDX3X for activating interferon signaling pathways and other antiviral mechanisms, establishing multilayered defense networks. This review systematically elaborates the functional diversity exhibited by DDX3X protein in virus interaction networks. DDX3X orchestrates viral genomic RNA processing during replication. Simultaneously, it interacts with host restriction factors to evade antiviral immunity, establishing a dynamic balance between viral propagation and host defense. The functional plasticity of DDX3X not only elucidates immune regulatory mechanisms in host-pathogen coevolution, but also provides novel molecular perspectives for deciphering zoonotic transmission barriers.

## 1 Introduction

**Figure 1:**
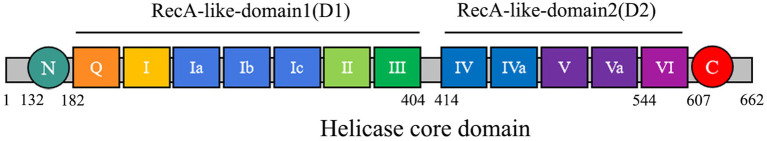
The core of DDX3X enzyme contains two RecA-like domains, namely Domain 1 and Domain 2.

Members of the DEAD-box helicase family play pivotal roles in RNA metabolism and cellular signaling regulation. As a crucial member of this family, the X-linked DEAD-box helicase 3 protein (DDX3X) not only participates in fundamental biological processes including RNA transcription, splicing, and translation, but also plays critical roles in cellular stress responses, disease pathogenesis, and viral replication. The multifunctional properties of DDX3X are closely associated with its characteristic molecular architecture (Figure 1) (Mo et al., 2021; De Colibus et al., 2022). The spatial configuration of these two domains collectively establishes specific binding pockets for RNA and ATP. Functional studies reveal that ATP binding and hydrolysis activities of DDX3X primarily depend on motifs Q, I, II, and VI, whereas its RNA-binding capacity is mediated by motifs Ia, Ib, Ic, IV, Iva, V, and VI (Shikalov et al., 2021). Notably, motifs III and Iva serve as critical signaling hubs bridging RNA and ATP binding sites. This precise structural partitioning and functional specialization enable DDX3X to efficiently execute its diverse biological roles (Park and Oh, 2022).

The innate immune system serves as the host’s primary defense against pathogens, operating through rapid detection of pathogen-associated molecular patterns and subsequent induction of effector molecules such as interferons (IFNs). IFNs establish a broad-spectrum antiviral state by activating downstream signaling cascades while coordinating adaptive immune responses. Recent advances highlight DDX3X as a critical regulator in immune networks, leveraging its RNA helicase and ATPase activities to modulate antiviral signaling (Swarup and Bolger, 2024; Kwon et al., 2022). Mechanistically, following viral RNA recognition by intracellular pattern recognition receptors RIG-I or MDA5, DDX3X is recruited to the mitochondrial antiviral signaling (MAVS) signalosome, where it facilitates the activation of interferon regulatory factor 3 (IRF3) and nuclear factor κB (NF-κB), ultimately driving transcriptional activation of interferon-*β* (IFN-β) (Oshiumi et al., 2010). This process involves dynamic interactions between DDX3X and key signaling molecules, including TRAF3 and TBK1. Beyond IFN signaling, DDX3X extensively engages with diverse immune regulatory networks. For instance, in Toll-like receptor signaling cascades, DDX3X-containing stress granules are dynamically disassembled via IKK complex-mediated phosphorylation, thereby releasing translational repression and enabling inflammatory responses (Samir et al., 2021). In the context of NLRP3 inflammasome activation, DDX3X may potentiate inflammatory responses through regulating reactive oxygen species homeostasis or potassium efflux. However, viruses and hosts have forged intricate co-evolutionary dynamics through persistent molecular arms races. While retaining evolutionarily conserved core biological functions such as RNA helicase activity, ATPase catalysis, and translational regulation, DDX3X exhibits phylogenetically diversified antiviral properties driven by structural disparities in immune systems across species. This functional divergence endows DDX3X with dual evolutionary characteristics during viral pathogenesis across species: conserved molecular functionality alongside lineage-specific adaptations observed in humans, artiodactyls, avians, and teleost species.

## 2 Viral interaction with DDX3X

### 2.1 Interactions between DDX3X and human viruses

Human viral pathogens, leveraging host specificity, genetic plasticity, and diversified transmission modalities, persistently constitute major public health threats. This is exemplified by herpesviruses-mediated lifelong latent infections, flavivirus-driven arthropod-borne diseases, and coronavirus-induced acute respiratory syndromes, collectively underscoring the multidimensional challenges in viral disease containment. During the infection cycles of these pathogens, DDX3X directly facilitates viral replication and particle assembly by interacting with viral RNA and structural proteins. Concurrently, it reprograms host immune surveillance networks to enable strategic evasion of antiviral responses. This functional plasticity positions DDX3X as a central hub for viral co-option of host networks, while simultaneously providing a molecular foundation for developing broad-spectrum, host-directed antiviral strategies (Table 1).

**Table 1:** DDX3X and human viruses.

No.	Virus	Mechanism	Function	Targeted cells/hosts
1	HSV-1	HSV-1 infection induces the direct interaction between DDX3X and pUL31, facilitating virus budding.	HSV-1 infection suppresses the expression of DDX3X in 143B cells. DDX3X promotes HSV-1 replication.	HeLa, Vero, 143B
2	EBV	miR-BART17-3p encoded by EBV targets and inhibits the expression of DDX3X.	DDX3X inhibits virus replication through the RLR pathway, and its abnormal function leads to persistent viral infection.	NKYS, T and NK cells of EBV patients
3	HCMV	HCMV infection depends on pp65 to integrate DDX3X into virions.	HCMV infection upregulates the expression of DDX3X, promoting virus spread and replication.	HFFs
4	HCV	HCV infection induces the phosphorylation of DDX3X, facilitating its binding to NS5A.	DDX3X promotes HCV replication.	Huh-7.5.1, Ava5, HEK293
5	ZIKV	DDX3X directly binds to the 5′ terminal region (TR) of ZIKV and exerts helicase activity.		In vitro experiments (helicase assay)
6	JEV	DDX3X binds to the 5′ untranslated region of JEV to positively regulate viral translation.		In vitro experiments (helicase assay)
7	DENV	The interaction between the DENV capsid protein and DDX3X downregulates the expression of DDX3X in the late stage of infection.	The expression of DDX3X is downregulated in the late stage of viral infection. DDX3X inhibits DENV replication.	HEK293T, Huh-7
8	HIV-1	DDX3X directly interacts with viral proteins such as Rev., Tat, Gag and viral RNA elements.	DDX3X promotes the translation of viral transcripts and virus assembly.	HEK293T, Huh-7, T
		Host hsa-miR-181-5p significantly downregulates the expression level of DDX3X by targeting the 3′-untranslated region of DDX3X mRNA.	Host hsa-miR-181-5p downregulates the expression of DDX3X to inhibit virus replication.	Jurkat, H9, H9-IIIB, 293 T
		DDX3X specifically recognizes abnormal short - chain RNAs generated during HIV-1 infection.	DDX3X activates IFN-I through MAVs and initiates the immune response of DC cells.	
		DDX3X inhibitors RK-33, FH-1321 and 6b block the ATPase or RNA - binding domain of DDX3X.	DDX3X inhibitors can inhibit virus replication and also have the potential to reverse latent infection.	Jurkat, J-Lat 11.1
9	SARS-CoV-2	The N protein of SARS-CoV-2 recruits DDX3X to the viral replication - transcription complex.	DDX3X promotes virus replication.	A549
		The N protein of SARS-CoV-2 releases DDX3X from antiviral stress granules.	DDX3X promotes viral RNA translation.	Vero E6, Calu-3
		DDX3X promotes the transcriptional level of the TMPRSS2 gene.	DDX3X inhibitors can reduce the viral load of SARS-CoV-2.	Calu-3
10	CV-B	DDX3X unwinds the secondary structure of the CV-B IRES.	DDX3X promotes viral protein synthesis.	Vero
11	HBV	HBV Pol interacts with IKKε, competitively blocking the binding of IKKε to DDX3.	HBV Pol inhibits the initiation of the DDX3-IKKε - mediated IFN-I signaling pathway to achieve immune escape.	HepG2, Huh7
		DDX3 inhibits cccDNA - dependent HBV RNA synthesis.		HepG2

**Figure 2:**
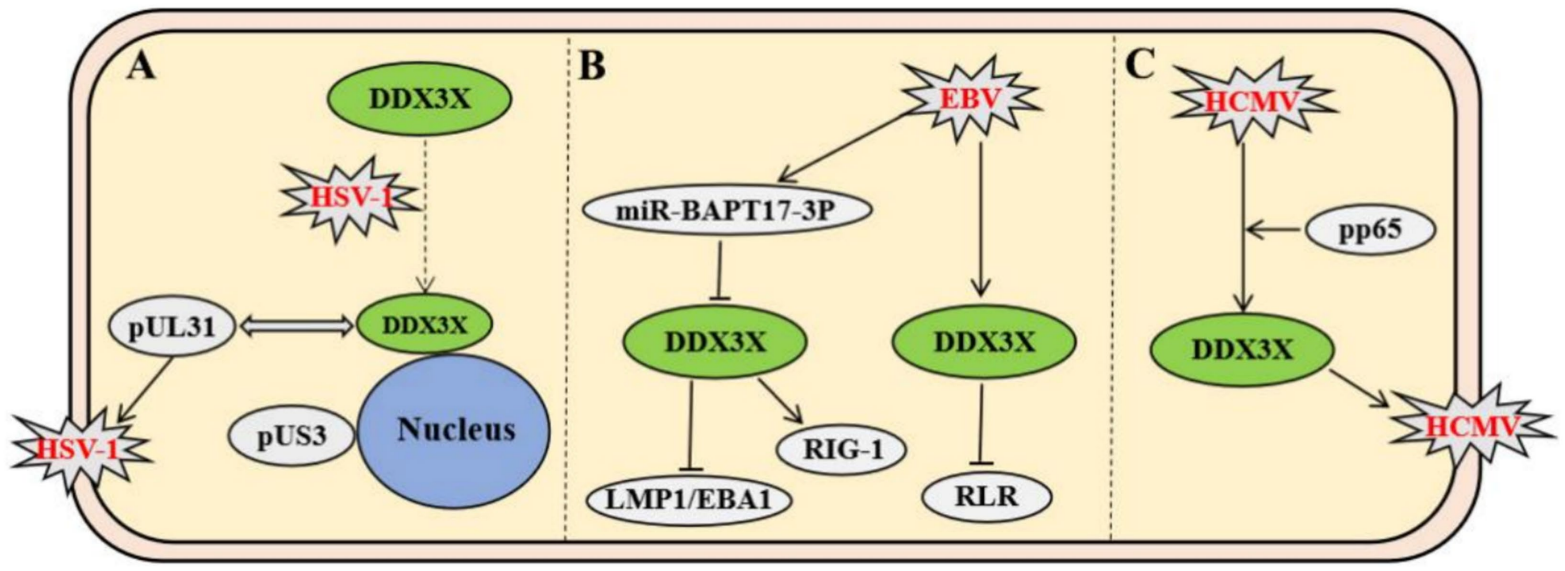
Virus-specific mechanisms of *Herpesviridae* members modulating host DDX3X protein. **(A)** HSV-1 recruits DDX3X to the nuclear envelope via interaction with the nuclear egress complex component pUL31, promoting C-capsid maturation and budding. Subsequently, the viral kinase pUS3 interacts with DDX3X to modulate cytoplasmic viral particle release. **(B)** EBV-encoded miR-BART17-3P suppresses DDX3X expression, impairing RIG-I-mediated RLR signaling and promoting expression of latency-associated proteins LMP1/EBNA1. DDX3X mutations block interferon activation downstream of RLR signaling. **(C)** HCMV tegument protein pp65 upregulates DDX3X expression and recruits it into virions, enhancing intercellular spread of mature virus.

Among members of the *Herpesviridae* family, the regulatory mechanisms of DDX3X exhibit remarkable virus-specific characteristics. In herpes simplex virus type 1 (HSV-1), DDX3X controls the entire viral gene expression cycle via its incorporation into mature virions. Both depletion and overexpression of DDX3X significantly suppress HSV-1 transcription and translation, leading to impaired viral genome replication and reduced virion assembly efficiency. This regulatory process is independent of its interferon-mediated pathway but dependent on its ATPase/helicase activity (Gringhuis et al., 2017). Further studies revealed that HSV-1 infection recruits DDX3X to the nuclear membrane. The interaction involves the carboxyl-terminal domain of DDX3X and pUL31, a component of the viral nuclear egress complex. This process facilitates the budding maturation of C-capsids. Concurrently, DDX3X interacts with viral kinase pUs3 and facilitates its incorporation into perinuclear virions to regulate cytoplasmic virion release. Functional impairment of DDX3X results in nuclear membrane retention of viral particles and significantly diminished extracellular virus production (Figure 2A) (Khadivjam et al., 2023). In contrast to HSV-1, which directly hijacks the enzymatic activity of DDX3X, Epstein–Barr virus (EBV) suppresses DDX3X expression via its encoded miR-BART17-3p. This downregulation weakens antiviral signaling through the host RIG-I-like receptor (RLR) pathway and promotes the expression of viral proteins LMP1/EBNA1 in NK cells to sustain latent infection (Jin et al., 2023). Clinical evidence indicates that patients with EBV-associated T/NK-cell lymphoproliferative disorders exhibit significantly elevated viral loads due to genetic or somatic DDX3X mutations, which impair interferon activation downstream of the RLR pathway; these alterations correlate with poor prognosis (Figure 2B) (Luo et al., 2023). Human cytomegalovirus (HCMV) upregulates DDX3X expression and recruits it into virions via the tegument protein pp65, facilitating the spread of mature HCMV. Knockdown of DDX3X markedly inhibits cell-to-cell transmission of clinical isolates (Figure 2C) (Cavignac et al., 2015).

**Figure 3:**
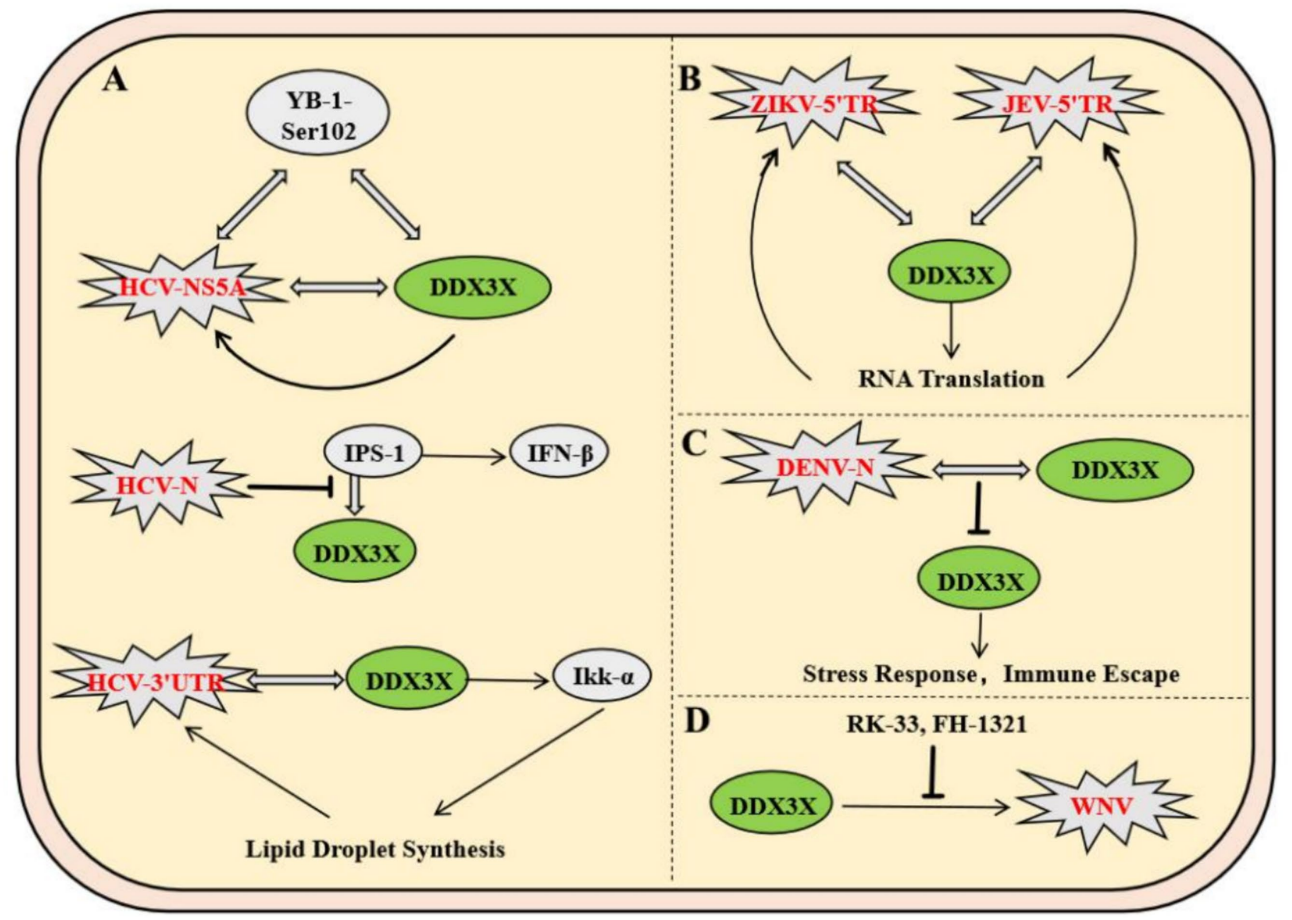
Molecular mechanisms of DDX3X exploitation by the *Flaviviridae* family and therapeutic targeting strategies. **(A)** NS5A forms a dynamic complex with the host factor YB-1-Ser102 to stabilize viral RNA replication, while DDX3X maintains viral RNA homeostasis. **(B)** DDX3X directly binds to the ZIKV RNA 5′-end to resolve secondary structures or interacts with the JEV 5′UTR to facilitate translation. It also collaborates with NS3/NS5 to form a replication complex dependent on its helicase activity. **(C)** The capsid protein downregulates DDX3X via its N-terminal domain to mediate immune evasion. **(D)** Small-molecule inhibitors RK-33 and FH-1321 specifically inhibit DDX3X activity, blocking viral protein translation.

*Flaviviridae* viruses have universally evolved molecular mechanisms to exploit the host DDX3X protein, with hepatitis C virus (HCV) being the most extensively studied representative within this family. In HCV, the nonstructural protein NS5A and the host factor YB-1 form a dynamic complex with DDX3X. Phosphorylation of YB-1 at Ser102 facilitates its interaction with NS5A. This binding stabilizes NS5A and modulates its phosphorylation state, ultimately enhancing viral RNA replication and infectious particle production. Meanwhile, DDX3X primarily contributes to the maintenance of steady-state viral RNA replication (Wang et al., 2015). Notably, in uninfected cells, DDX3X binds to the CARD-like domain of the mitochondrial antiviral signaling protein IPS-1 via its C-terminal region, localizes to perimitochondrial regions, and enhances IPS-1-mediated IFN-*β* signaling activation (Figure 3A) (Oshiumi et al., 2010). Similar to HCV, Zika virus (ZIKV) and Japanese encephalitis virus (JEV) have evolved DDX3X-dependent molecular mechanisms to enhance their replication. In ZIKV infection, DDX3X directly binds to the 5′ terminal region of the viral RNA, exerting helicase activity to unwind secondary structures critical for replication. During JEV infection, DDX3X interacts with the 5′ untranslated region (UTR) of the viral genome to positively regulate RNA translation. Concurrently, DDX3X colocalizes with JEV nonstructural proteins NS3 and NS5 to form replication complexes, which depend on DDX3X to maintain enzymatic activity for efficient viral genome synthesis (Figure 3B) (Nelson et al., 2021). In contrast to the aforementioned viruses, DDX3X exhibits antiviral functions during dengue virus (DENV) infection. Genetic silencing of DDX3X significantly elevates DENV titers, whereas its overexpression suppresses viral replication through a non-interferon-dependent stress response pathway (Kumar et al., 2017). Mechanistic studies demonstrate that the DENV capsid protein interacts with the amino acid residues 223–350 of DDX3X via its N-terminal domain, downregulating DDX3X expression in late infection to achieve immune evasion (Figure 3C) (Brai et al., 2022). In West Nile virus (WNV) infection, DDX3X activity can be specifically inhibited by small-molecule compounds RK-33 and FH-1321. These inhibitors disrupt viral protein translation at post-entry stages, markedly reducing replication efficiency, a mechanism potentially linked to DDX3X’s roles in viral RNA unwinding or regulation of host translation machinery (Figure 3D) (Brai et al., 2019). Notably, these inhibitors not only display favorable pharmacokinetic profiles and safety but also provide a differentiated targeting rationale for broad-spectrum antiviral drug development, owing to WNV’s lack of direct DDX3X exploitation for immune evasion.

**Figure 4:**
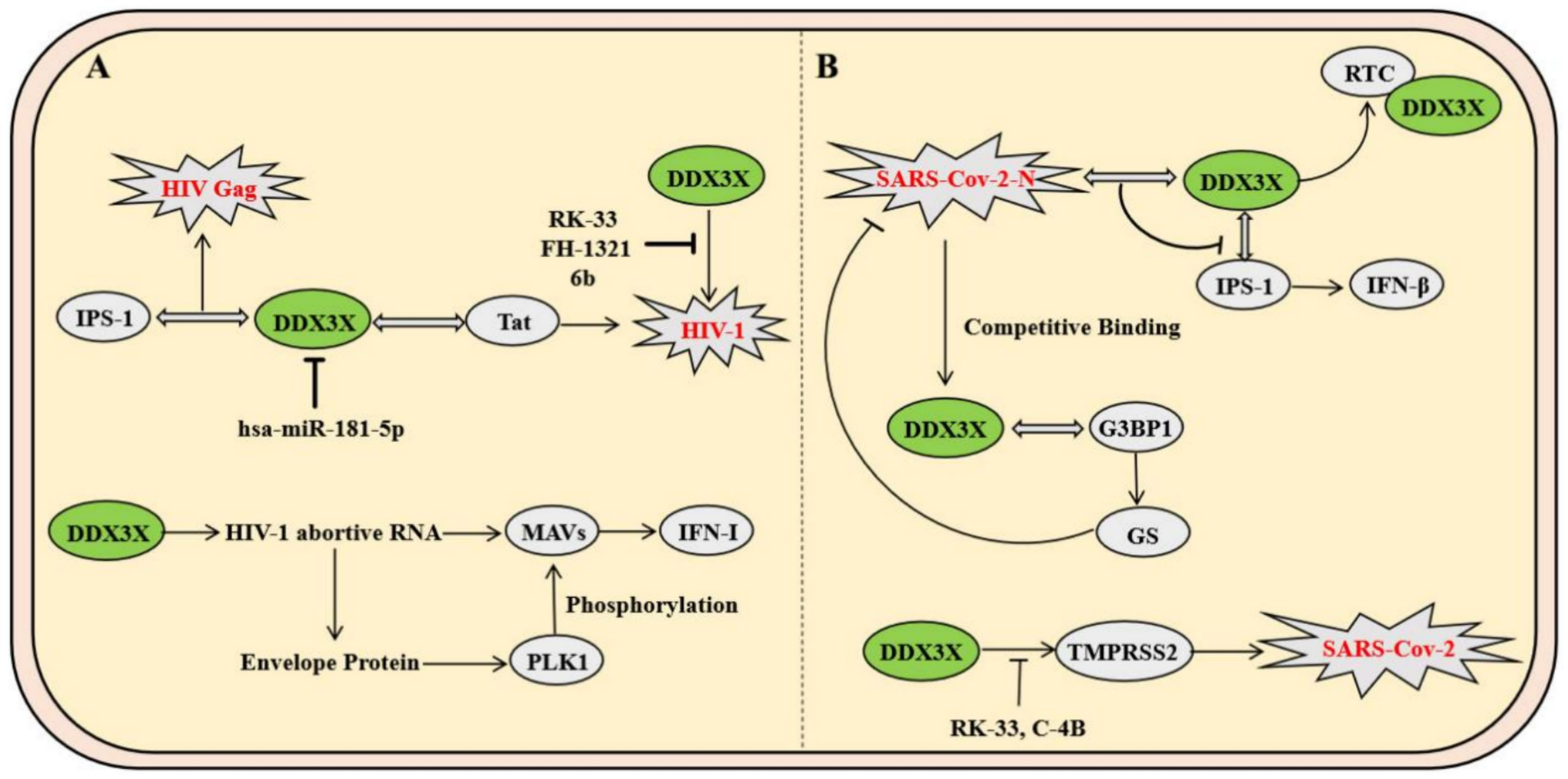
Mechanisms of DDX3X in HIV-1 and SARS-CoV-2 infection. **(A)** DDX3X promotes viral replication by binding to IPS-1 and Tat, while host hsa-miR-181-5p downregulates its expression. Additionally, DDX3X facilitates phosphorylation of the viral envelope protein through PLK1 and activates the MAVS pathway to enhance viral propagation. **(B)** The SARS-CoV-2 N protein interacts with DDX3X to suppress its helicase activity and block IFN-*β* signaling by disrupting its interaction with IPS-1. Conversely, DDX3X resolves G-quadruplex structures in the TMPRSS2 promoter to promote transcription, and pharmacological inhibitors targeting DDX3X reduce TMPRSS2 expression, thereby inhibiting viral entry.

As a major global public health threat, human immunodeficiency virus type 1 (HIV-1) pathogenesis is characterized by persistent immune system destruction and latent infection. DDX3X serves as a critical host factor in HIV-1 replication, participating in the viral life cycle through multiple mechanisms. Firstly, DDX3X binds to the Rev. protein and Rev. response element, forming a viral RNA export complex that significantly enhances the translation efficiency of the viral structural protein Gag and facilitates virion assembly (Heaton et al., 2023). Secondly, DDX3X directly interacts with the viral transcriptional activator Tat and its target TAR hairpin structure, boosting the translation of viral transcripts while potentially suppressing host antiviral immunity by inhibiting the MAVS signaling pathway. However, hosts have evolved sophisticated regulatory networks to counteract this process. For instance, hsa-miR-181-5p downregulates DDX3X expression by targeting its 3′-untranslated region (3′UTR), impairing viral RNA nuclear export and reducing production of the capsid protein P24 (Han et al., 2023). At the innate immunity level, DDX3X participates in early host antiviral defense mechanisms. Mechanistically, DDX3X specifically recognizes abortive RNA generated during HIV-1 infection, activating the type I interferon signaling pathway via the MAVS, thereby inducing dendritic cell maturation and initiating adaptive immune responses (Gringhuis et al., 2017). However, HIV-1 counteracts this defense by engaging its envelope glycoprotein with the C-type lectin receptor DC-SIGN on dendritic cells, which triggers PLK1 kinase-mediated phosphorylation of the MAVS complex, ultimately suppressing interferon-stimulated gene (ISG) expression to achieve immune evasion. In antiviral drug development, DDX3X-specific inhibitors such as RK-33, FH-1321, and the novel compound 6b block DDX3X’s ATPase or RNA-binding domains, demonstrating dual efficacy in suppressing viral replication and reversing latent infection (Rao et al., 2021). Notably, compound 6b exhibits broad-spectrum antiviral activity *in vivo*, showing potent inhibition against multidrug-resistant HIV-1 strains (Brai et al., 2020). These findings underscore that targeting the DDX3X regulatory network not only inhibits viral replication but also restores host immune responses, offering a dual therapeutic strategy for HIV treatment (Figure 4A).

Severe acute respiratory syndrome coronavirus 2 (SARS-CoV-2), a representative member of the Betacoronavirus genus, relies critically on its nucleocapsid protein for viral lifecycle progression. Mechanistic studies reveal that the SARS-CoV-2 N protein specifically interacts with the host RNA helicase DDX3X through its N-terminal RNA-binding domain, recruiting DDX3X to the viral replication-transcription complex. This interaction not only enhances the binding affinity of the N protein to double-stranded RNA but also suppresses DDX3X’s RNA helicase activity. Furthermore, the N-DDX3X complex disrupts DDX3X-IPS-1 binding, inhibiting the IFN-*β* signaling pathway and hijacking DDX3X to viral replication sites to promote viral propagation (Lodola et al., 2023). Notably, SARS-CoV-2 employs its N protein to competitively bind stress granule scaffolding proteins such as G3BP1, thereby suppressing stress granule assembly. This process liberates DDX3X from antiviral stress granules, redirecting it to support viral RNA translation (Ciccosanti et al., 2021). Another study demonstrates that DDX3X regulates host TMPRSS2 expression by resolving G-quadruplex structures in the TMPRSS2 promoter region to enhance its transcription. The small-molecule inhibitor RK-33 significantly reduces TMPRSS2 levels through DDX3X inhibition, thereby blocking the protease-dependent pathway of viral cellular entry (Vesuna et al., 2022). Furthermore, DDX3X-specific inhibitors RK-33 and C-4B effectively reduce SARS-CoV-2 viral loads in Calu-3 and Vero E6 cells, with consistent efficacy against Alpha, Beta, and Delta variants (Figure 4B) (Vesuna et al., 2022).

**Figure 5:**
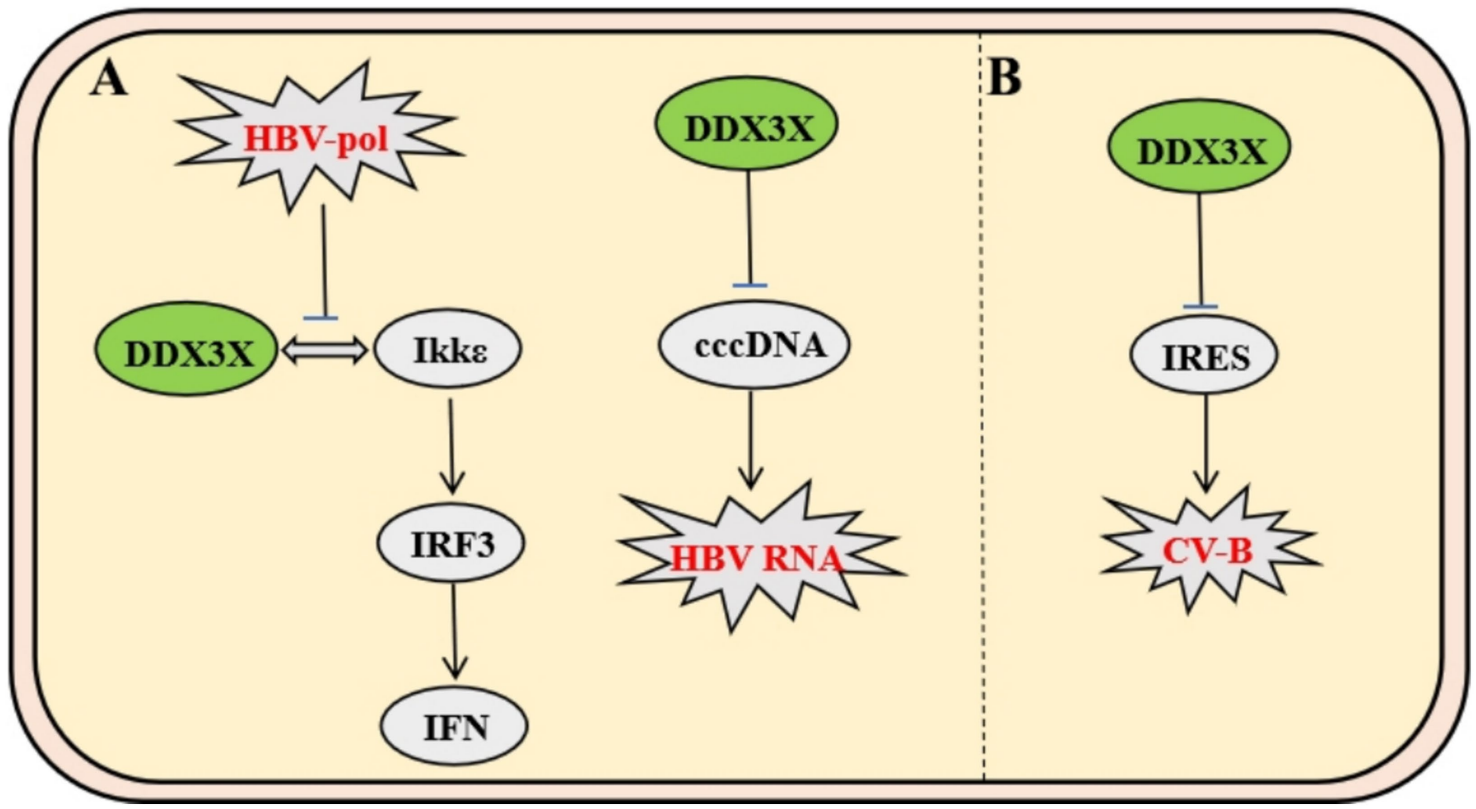
Mechanisms of DDX3X in HBV and CV-B infection**. (A)** HBV polymerase binds IKKε via its C-terminal domain, competitively disrupting the IKKε-DDX3X interaction. This inhibits TBK1/IKKε complex activation and IRF3 phosphorylation, enabling evasion of IFN-I signaling. Separately, DDX3X suppresses cccDNA-dependent HBV RNA synthesis. **(B)** CV-B utilizes IRES-dependent translation, where DDX3X resolves IRES secondary structures to facilitate viral protein synthesis. Specific inhibitors targeting DDX3X suppress CV-B replication by disrupting this mechanism.

The hepatitis B virus (HBV) polymerase interacts specifically with the host immune signaling kinase IKKε through its unique structural domains. HBV Pol forms a stable complex with the kinase domain of IKKε via its terminal protein domain, competitively blocking the physiological interaction between IKKε and DDX3X (Wang and Ryu, 2010). This steric hindrance suppresses TBK1/IKKε activation, impairing IRF3 phosphorylation and nuclear translocation, thereby disrupting DDX3X-IKKε-mediated IFN-I signaling initiation and enabling immune evasion (Jin et al., 2023). Subsequent studies revealed that DDX3X also significantly inhibits cccDNA-dependent HBV RNA synthesis, an effect independent of its interaction with HBV Pol or the X protein (Ko et al., 2014). These findings demonstrate that DDX3X not only serves as an innate immune activator hijacked by viruses but also exerts antiviral functions through epigenetic regulatory mechanisms (Figure 5A).

Coxsackievirus B (CV-B), a member of the *Picornaviridae* family, relies on a highly structured internal ribosome entry site (IRES) within its 5′ untranslated region (5′UTR) for genomic translation. Studies demonstrate that DDX3X facilitates viral protein synthesis by unwinding secondary structures in the IRES. DDX3X inhibitors significantly suppress CV-B replication while exhibiting no significant effect on measles virus, which depends on the 5′ cap-dependent translation mechanism. This highlights DDX3X’s specific regulatory role in IRES-dependent viral translation and provides a novel rationale for developing broad-spectrum antiviral agents targeting non-canonical translation pathways in IRES-driven viruses (Figure 5B) (Quaranta et al., 2020).

### 2.2 Interactions between DDX3X and animal viruses

Besides human viruses, DDX3X exhibits unique evolutionary adaptability in animal virus-host interactions. The virus-host interaction mechanism is critically dependent on viral evolutionary traits and host adaptability. Animal and human viruses exhibit fundamental differences in immune interaction mechanisms, host adaptive evolution, and cross-species transmission strategies, which are deeply rooted in the long-term synergistic co-evolution among host ecological niches, physiological architectures, and immune systems (Table 2).

**Table 2:** DDX3X and animal viruses.

No.	Virus	Mechanism	Function	Targeted cells/hosts
1	FMDV	DDX3X specifically interacts with RPL13 to form a DDX3X - RPL13 - IRES ternary complex, enhancing the binding of IRES to eIF3e/eIF3j.	DDX3X promotes FMDV replication. FMDV 3C pro degrades DDX3X to weaken the host immune response.	PK-15
2	PRRSV	PRRSV infection induces the expression of DDX3X, driving ferroptosis.	PRRSV infection promotes the expression of DDX3X. DDX3X promotes virus replication.	Marc-145
3	VACV	The K7 protein of VACV specifically binds to DDX3X to inhibit the transcription of IFN-β.	The specific binding of the VACV K7 protein to DDX3X significantly enhances virus replication.	L929, Hela, BALB/c mice
4	PRV	PRV hijacks DDX3X to drive KIF1A - mediated anterograde axonal transport of the virus and cause IL-6 - dependent immunosuppression.	PRV hijacks DDX3X to activate the neuronal opioid receptor signaling pathway and promote virus spread.	SCG, PK-15
5	NDV and AIV	chDDX3X activates the chSTING - chTBK1 - chIRF7 - IFN-β signaling axis to inhibit the replication of NDV.	Virus infection upregulates chDDX3X, which activates IFN to inhibit the replication of NDV and AIV.	DF-1
6	TMUV	duDDX3X interacts with NS2A and regulates interferon through the TBK1 - dependent pathway.	TMUV inhibits the expression of duDDX3X to promote virus replication.	BHK-21
7	SHVV	The P protein of SHVV binds to DDX3X to maintain the stability of the P protein.	SHVV infection promotes the expression of DDX3X. DDX3X promotes virus replication.	CCO
8	RGNNV	RGNNV hijacks EcDDX3X to activate the IRF3/7 signaling axis and drive antiviral effects such as IFN-β.	EcDDX3X inhibits the transcription of viral RNA polymerase and capsid protein.	GS
9	LASV JUNV	DDX3 directly binds to the nucleoprotein NP of arenaviruses, inhibiting the host IFN-I pathway and weakening the innate immune response.	DDX3 assists vRNP to significantly enhance the efficiency of viral RNA replication and transcription.	A549
10	VEEV	The nsP3 of VEEV interacts with DDX3 to recruit eIF4A, eIF4G and PABP, promoting viral mRNA translation.	DDX3 promotes VEEV replication.	U87MG
11	MCMV	MCMV m139 forms a complex with DDX3X and UBR5, blocking the activation of the DDX3X - mediated IFN-α/β signaling pathway.	DDX3X promotes MCMV replication.	SVEC4-10, iBMDM, 10.1 fibroblasts

**Figure 6:**
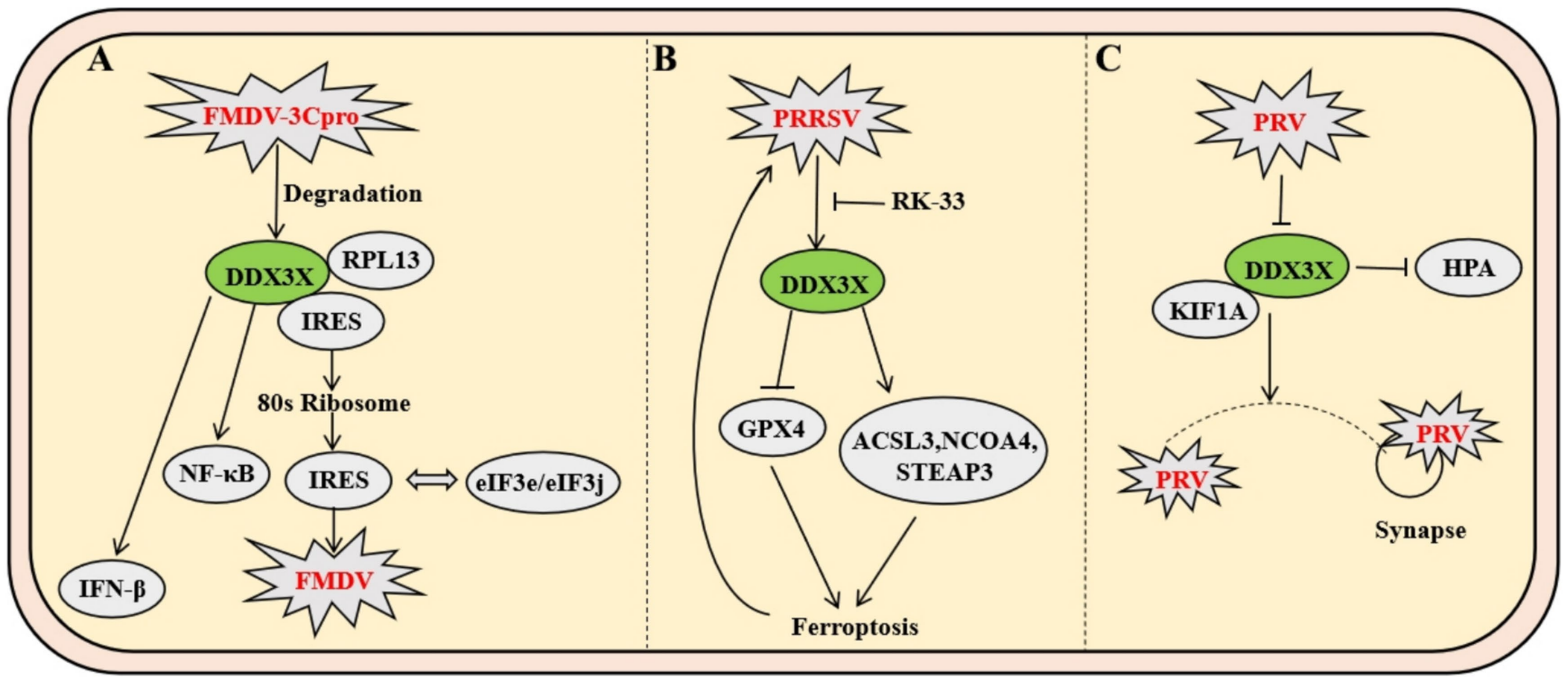
Mechanisms of DDX3X in FMDV and PRRSV infection. **(A)** FMDV utilizes IRES-mediated translation, where DDX3X forms a complex with RPL13 to enhance 80S ribosome recruitment and strengthen eIF3e/eIF3j-IRES binding, thereby promoting viral protein synthesis. Concurrently, the viral protease 3Cpro cleaves DDX3X to induce its degradation, impairing host antiviral responses by suppressing NF-κB and IFN-β signaling pathways. **(B)** PRRSV upregulates DDX3X expression, which facilitates viral replication by inducing ferroptosis through downregulation of GPX4 and upregulation of ACSL3, among other genes. This dysregulation of cellular redox balance creates a favorable environment for viral propagation. **(C)** PRV infection induces DDX3X translocation from neuronal soma to axons, where it interacts with kinesin KIF1A to facilitate anterograde viral particle transport.

As a prototypical member of the *Picornaviridae* family, foot-and-mouth disease virus (FMDV), a positive-sense single-stranded RNA virus, executes genomic translation through a highly structured internal ribosome entry site (IRES) rather than the conventional 5′ cap-dependent mechanism. Mechanistic studies reveal that during FMDV infection, DDX3X specifically interacts with RPL13 through its N-terminal domain to form a DDX3X-RPL13-IRES ternary complex. This macromolecular assembly significantly enhances IRES-mediated translational efficiency by orchestrating the precise recruitment of 80S ribosomal subunits and strengthening the binding affinity between IRES and eukaryotic initiation factors eIF3e/eIF3j, thereby driving the robust synthesis of both structural and nonstructural viral proteins (Guan et al., 2021). In antiviral defense, DDX3X orchestrates the activation of NF-κB and IFN-*β* signaling pathways to induce the expression of interferon-stimulated genes (ISGs) and proinflammatory cytokines (e.g., IL-6, TNF-*α*), thereby establishing a robust antiviral barrier. Conversely, FMDV subverts this defense via its nonstructural protein 3C protease, which executes site-specific cleavage of DDX3X, leading to proteolytic degradation and consequent attenuation of host immune responses. Genetic ablation of DDX3X significantly destabilizes the eIF3e/3j-IRES interaction, resulting in selective downregulation of viral protein synthesis rates. Notably, RPL13 depletion specifically impairs DDX3X-mediated assembly of the translation initiation complex. The stringent specificity of the DDX3X-RPL13 interaction suggests evolutionary conservation of DDX3X’s functional role in IRES-dependent viral translation. These findings elucidate the dynamic equilibrium between RNA helicases and ribosomal proteins in virus-host antagonism, providing a mechanistic framework for developing translation-targeted antiviral therapeutics (Figure 6A) (Guan et al., 2021).

As a pivotal member of the *Arteriviridae* family, porcine reproductive and respiratory syndrome virus (PRRSV) infection induces marked upregulation of DDX3X protein expression in Marc-145 cells. The upregulation of DDX3X facilitates viral replication. Integrated transcriptomic and metabolomic analyses demonstrate that DDX3X gene silencing in PRRSV-infected cells profoundly dysregulates critical biological processes, including ferroptosis, FoxO signaling, and glutathione metabolism. Subsequent transmission electron microscopy imaging revealed that PRRSV-infected cells exhibit mitochondrial ultrastructural hallmarks characteristic of ferroptosis, a phenomenon ameliorated by the DDX3X-specific inhibitor RK-33 or si-DDX3X intervention. Ferroptosis-associated factor profiling demonstrated PRRSV-mediated transcriptional downregulation of the antioxidant factor GPX4, concurrent with upregulated expression of pro-ferroptotic genes ACSL3, NCOA4, STEAP3. These transcriptional perturbations were reversed upon DDX3X silencing. Collectively, these findings establish that PRRSV-induced DDX3X expression drives ferroptotic cascades to create a proviral microenvironment conducive to viral replication (Figure 6B) (Mao et al., 2024).

Pseudorabies virus (PRV), a neurotropic alphaherpesvirus, exploits the host RNA helicase DDX3X to enhance its anterograde axonal spread. Infection triggers DDX3X downregulation and relocalization from neuronal somata into axons, where it interacts with microtubule-associated proteins, including the anterograde motor KIF1A, to directly facilitate viral particle transport towards synaptic terminals. This dependency is critical for wild-type PRV but absent in the attenuated Bartha strain due to its deficient US9-gE-gI complex, which fails to recruit DDX3X. Genetic or pharmacological inhibition of DDX3X selectively blocks PRV anterograde spread without impairing somatic replication. Furthermore, DDX3X ablation *in vivo* causes selective loss of small-diameter sensory neurons, leading to compulsive scratching; this neuropathic injury induces IL-6 and G-CSF release, activating the HPA axis, elevating systemic corticosterone, driving thymic atrophy, impairing T-cell development, and collectively compromising antiviral immunity (Figure 6C) (Cronin et al., 2023).

**Figure 7:**
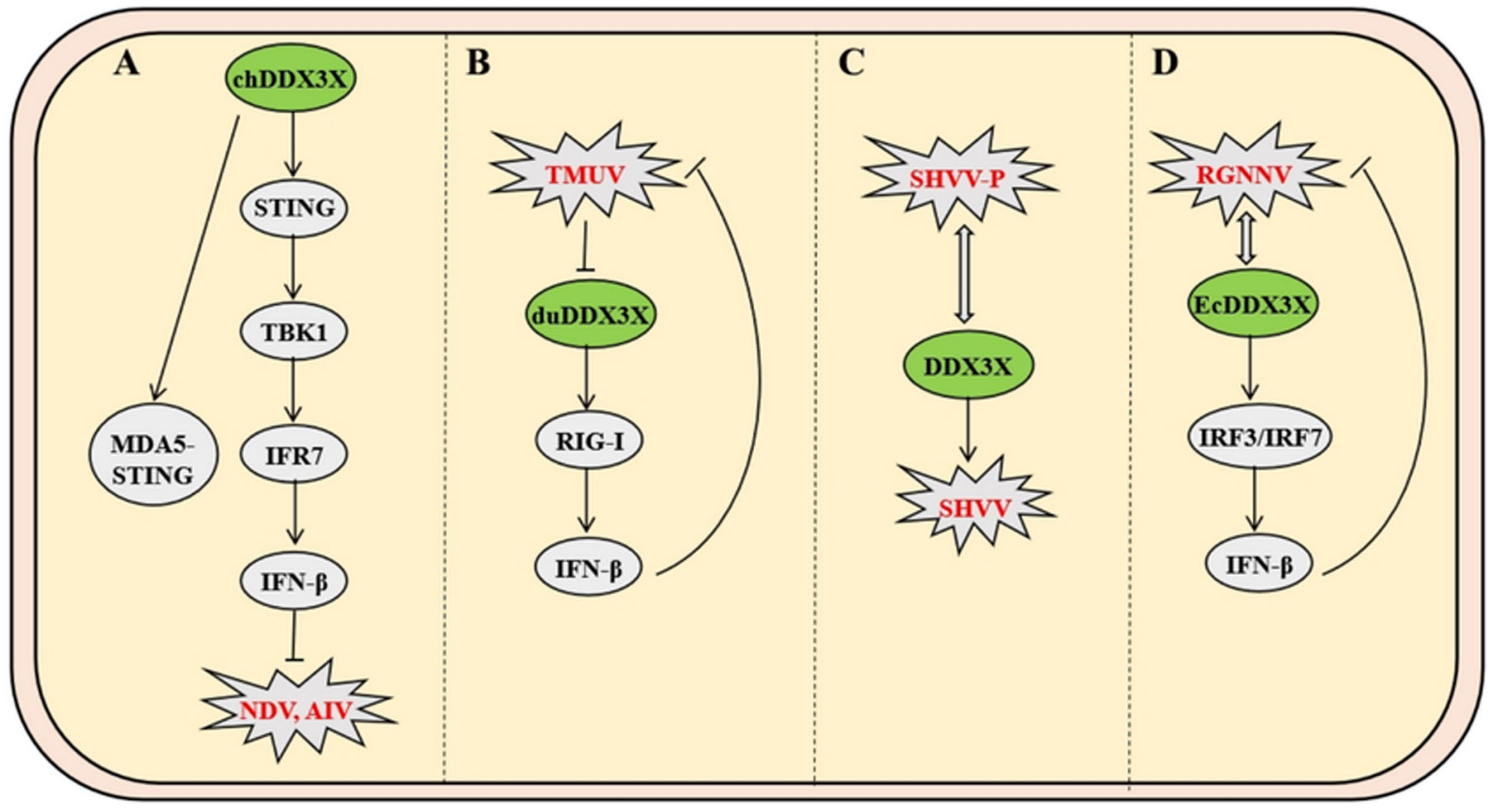
Mechanisms of DDX3X in avian and fish viral infections. **(A)** Avian chDDX3X upregulates STING to promote TBK1 phosphorylation and IRF7 activation, thereby activating the MDA5-STING-IFN-β axis to suppress Newcastle disease virus (NDV) and avian influenza virus. **(B)** TMUV infection downregulates duck duDDX3X expression, which inhibits viral replication through RIG-I-mediated suppression of IFN-β production. **(C)** The SHVV P protein binds to the N-terminal and core domains of DDX3X, evading autophagic degradation to stabilize P protein and sustain infection. **(D)** RGNNV interacts with EcDDX3X to activate IRF3/IRF7 signaling, which paradoxically restricts viral replication by enhancing host antiviral responses.

Unlike mammals, avian species lack typical lymph node structures and primarily rely on diffusely distributed lymphoid tissues in the digestive and respiratory systems for peripheral immune responses. As a unique central immune organ in birds, the bursa of Fabricius plays an indispensable role in the differentiation and maturation of B lymphocytes. Newcastle disease (ND) and avian influenza (AI) are two major infectious diseases threatening avian health, causing mass mortality and significant economic losses. Studies show that overexpression of chicken DDX3X (chDDX3X) in DF-1 cells upregulates transcription of IFN-*β*, IL-1β, IL-6, IL-8, and interferon-stimulated genes (ISGs), while exerting dose-dependent inhibition on Newcastle disease virus (NDV) and avian influenza virus (AIV) replication. Conversely, siRNA-mediated chDDX3X knockdown reduces IFN-β production and promotes viral replication. Avian species have evolved a unique pattern recognition network due to the absence of RIG-I receptor. Chicken MDA5 specifically binds to the endoplasmic reticulum transmembrane protein STING, forming an MDA5-STING-IFN-*β* signaling pathway distinct from the mammalian MAVS/RIG-I complex (Cheng et al., 2015). Mechanistically, chDDX3X induces antiviral IFN-β secretion by upregulating STING protein levels, promoting TBK1 phosphorylation, and activating IRF7 transcription factor. These findings demonstrate that chDDX3X restricts NDV replication by activating the chSTING-chTBK1-chIRF7-IFN-β signaling axis (Niu et al., 2019). Compared with AIV’s viral protein-mediated interference with host signaling during infection, chDDX3X exhibits stronger sustained inhibition of NDV replication, highlighting an evolved specific anti-RNA virus defense mechanism in avians (Figure 7A).

In duck models, Tembusu virus (TMUV) infection downregulates the expression of duDDX3X in duck embryo fibroblasts. Overexpression of duDDX3X suppresses TMUV replication at the early stage of infection by enhancing RIG-I-mediated IFN-β production (Li et al., 2020). Proteomic analysis reveals that in TMUV-infected BHK-21 cells, duDDX3X regulates key nodes of the interferon signaling pathway through a TBK1-dependent mechanism, with this regulatory effect involving direct interaction with the viral NS2A protein (Gu et al., 2017). In contrast to chDDX3X, duDDX3X exhibits weaker inductive capacity for proinflammatory cytokines (e.g., IL-1β and IL-6), and its antiviral effects primarily rely on the IFN-β-mediated immune regulatory network. This finding provides critical evidence for the functional diversity of avian DDX3X proteins (Figure 7B).

As the most primitive vertebrates, fish exhibit a mucosa-dominated tertiary immune defense system characterized by diffuse lymphoid tissues with cold-adaptation capabilities. In infection models of Snakehead vesiculovirus (SHVV), the viral phosphoprotein (P protein) binds directly to the N-terminal and core domains of host DDX3X, hijacking DDX3X to stabilize P protein by evading autophagic-lysosomal degradation, thereby enhancing viral replication. Overexpression of DDX3X amplifies SHVV P protein accumulation and viral titers, while competitive expression of its core domain disrupts DDX3X-P interaction and suppresses replication, a process independent of IFN pathways (Figure 7C) (Bei et al., 2023). Conversely, in Grouper nervous necrosis virus (RGNNV) infection, host EcDDX3X activates the IRF3/IRF7 axis via its N-terminal domain, potently upregulating interferon promoter activity and antiviral effectors. This antiviral response suppresses viral RNA polymerase and capsid protein transcription through IFN-dependent mechanisms (Figure 7D) (Liu et al., 2017).

**Figure 8:**
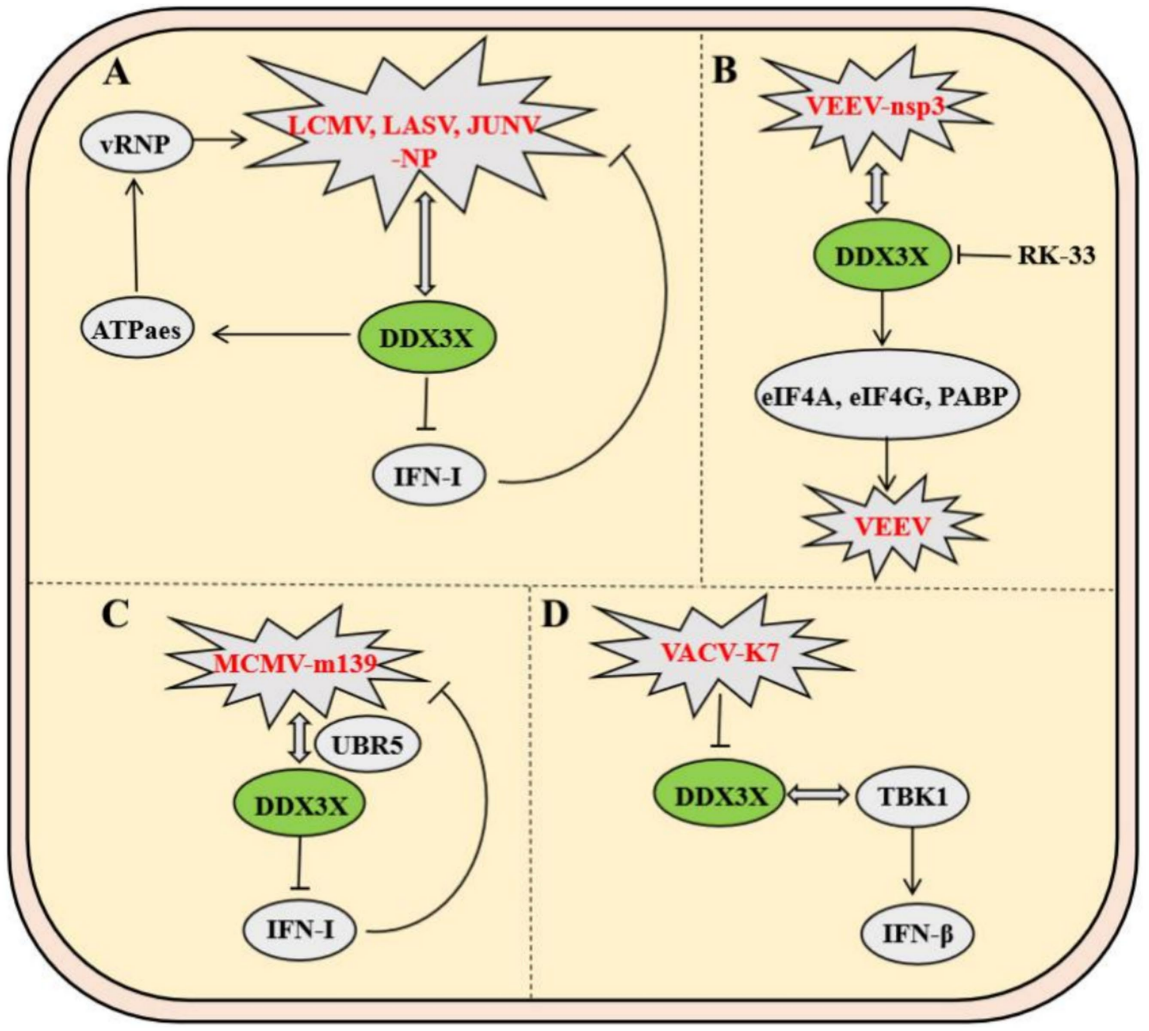
Mechanisms of DDX3X in arenavirus and poxvirus infections. **(A)** The arenavirus nucleoprotein (NP) binds DDX3X to inhibit type I interferon (IFN-I) signaling while promoting viral RNA replication. **(B)** The VEEV nsp3 protein interacts with DDX3X to facilitate viral replication by recruiting translation initiation factors eIF4A, eIF4G, and PABP. **(C)** MCMV m139 forms a complex with DDX3X and UBR5, suppressing IFN-*α*/β signaling to enhance viral replication in macrophages. **(D)** The vaccinia virus K7 protein binds the helicase domain of DDX3X, blocking its interaction with TBK1 to inhibit IFN-β activation.

Arenaviruses, enveloped insect viruses with characteristic granular inclusions, belong to Subgroup B of the family *Arenaviridae*. Mechanistic studies demonstrate that DDX3X promotes viral immune evasion by directly binding to nucleoprotein of multiple arenaviruses-including Lymphocytic choriomeningitis virus (LCMV), Lassa virus (LASV), and Junín virus (JUNV)-thereby suppressing host IFN-I production and impairing innate immunity. Furthermore, DDX3X enhances viral RNA replication and transcription efficiency through its ATPase/helicase activity, which facilitates the assembly and functionality of viral ribonucleoprotein (vRNP) complexes. Strikingly, this mechanism remains highly conserved across both Old World and New World arenaviruses, including hemorrhagic fever-associated LASV and JUNV, establishing DDX3X as a central hub for viral hijacking of host machinery. Notably, DDX3X exerts stage-dependent regulation: its proviral effects operate independently of IFN-I signaling during early infection, while later stages involve amplification of viral replication through interferon pathway suppression, revealing its multifaceted role in dynamically regulating host-virus interplay (Figure 8A) (Loureiro et al., 2018).

Venezuelan equine encephalitis virus (VEEV), a mosquito-borne New World alphavirus classified as a Select Agent Category B pathogen, critically depends on the host RNA helicase DDX3X for replication. Co-immunoprecipitation coupled with mass spectrometry revealed a direct interaction between VEEV nonstructural protein nsP3 and DDX3X, while DDX3X knockdown significantly reduced viral titers, identifying DDX3X as an essential host factor for VEEV propagation. Further mechanistic investigations demonstrated that the nsp3-DDX3X complex recruits eukaryotic translation initiation factors (eIF4A, eIF4G) and poly (A)-binding protein to assemble functional translation initiation complexes, thereby facilitating efficient viral mRNA translation. This highlights nsP3’s role in directly hijacking DDX3X to orchestrate viral replication. Notably, the small-molecule inhibitor RK-33, targeting the ATPase activity of DDX3X, disrupts its interactions with nsP3 and translational machinery, achieving potent antiviral effects. These findings delineate the central role of DDX3X in the VEEV life cycle and validate its potential as a host-directed therapeutic target (Figure 8B) (Amaya et al., 2016).

In murine models, Murine cytomegalovirus (MCMV) employs the viral protein m139 to target host DDX3X and the ubiquitin ligase UBR5, forming a ternary complex that collaboratively suppresses the IFN-I response—thereby evading host antiviral immunity. The m139-DDX3X-UBR5 complex impedes DDX3X-mediated activation of the IFN-*α*/*β* signaling pathway while leveraging UBR5-dependent functions to facilitate efficient viral replication in endothelial cells and macrophages. *In vitro* and *in vivo* experiments demonstrate that infection with an m139-deficient MCMV strain leads to significantly elevated host IFN expression in macrophages, effectively restricting viral dissemination in vivo. These findings not only elucidate a strategy by which MCMV escapes immune surveillance through DDX3X targeting but also highlight the critical role of the DDX3X-UBR5 interaction in regulating viral replication (Figure 8C) (Puhach et al., 2020).

Vaccinia virus (VACV) employs its early-expressed K7 protein to specifically bind the D/E-rich motif and helicase domain of DDX3X, inducing conformational remodeling that antagonizes DDX3X-TBK1 kinase interaction, thereby suppressing IFN-*β* transcriptional activation. By subverting this pivotal host antiviral signaling axis, the K7 protein potentiates VACV replication efficiency and dissemination capacity. Animal studies further validate that DDX3X-TBK1 complex formation critically governs viral pathogenicity in vivo (Figure 8D) (Benfield et al., 2013).

## 3 Conclusion and perspectives

As a core member of the DEAD-box RNA helicase family, DDX3X plays a pivotal role in viral life cycle regulation and host immune responses through its ATP-dependent nucleic acid helicase activity. Its evolutionarily conserved RNA helicase function renders it a critical host cofactor for multiple viruses. For example, human viruses including HSV-1, HCMV, HCV, HIV-1, and SARS-CoV-2 utilize DDX3X to facilitate viral RNA replication. Beyond its helicase activity, the FMDV in artiodactyls enhances IRES-mediated viral protein translation through the DDX3X-RPL13 complex. PRRSV promotes viral replication by upregulating DDX3X expression to drive ferroptosis. Additionally, while supporting viral replication, DDX3X’s innate immune regulatory functions profoundly influence pathogen immune evasion strategies, whereby viruses such as HCV, HBV, DENV, and HIV-1 commonly exploit DDX3X to subvert the interferon (IFN) pathway for immune evasion. Host adaptive evolution has further shaped DDX3X functional diversity. In avian species, the absence of the RIG-I receptor leads to reliance on the MDA5-STING antiviral pathway, driving NDV to evolve a strategy that activates IFN-β via the DDX3X-STING-TBK1 axis to restrict its own replication. As primitive vertebrates, fish exhibit distinct antiviral mechanisms. The *Siniperca chuatsi* rhabdovirus, classified within the *Rhabdoviridae* family and abbreviated as SHVV, evades autophagic-lysosomal degradation by stabilizing its phosphoprotein through DDX3X-mediated mechanisms. In contrast, the red-spotted grouper nervous necrosis virus, belonging to the *Nodaviridae* family and designated RGNNV, activates the IRF3/7 signaling axis via EcDDX3X dependency to induce interferon production. These divergent strategies exemplify family-specific viral exploitation of host machinery. Significantly, the ATPase-targeting small-molecule inhibitor RK-33 demonstrates broad-spectrum antiviral activity against multiple pathogens: human viruses such as WNV, HIV-1, and SARS-CoV-2; the artiodactyl pathogen PRRSV; and the arthropod-borne VEEV. This establishes DDX3X as a conserved host target across species. The coexistence of functional conservation and host-adaptive divergence in DDX3X interactions not only elucidates fundamental principles of viral host-factor exploitation but also creates a framework for developing cross-species antiviral therapeutics targeting this helicase.

Despite the promising potential of DDX3X-targeted antiviral strategies in recent years, several critical questions remain to be addressed. First, the molecular determinants underlying viruses’ specific selection of DDX3X domains or signaling pathways to achieve immune evasion or replication enhancement remain unclear. For instance, within the *Flaviviridae* family, dengue virus (DENV) and hepatitis C virus (HCV) exhibit opposing regulatory mechanisms on DDX3X—an observation suggesting that specific regions of the viral genome or protein interaction networks may play a decisive role. Second, although the DDX3X inhibitor RK-33 demonstrates broad-spectrum antiviral activity *in vitro*, it’s *in vivo* safety and impacts on host physiological functions require systematic evaluation. Given DDX3X’s involvement in RNA metabolism, cellular stress responses, and developmental regulation, long-term inhibition may elicit off-target effects. Additionally, functional divergence of DDX3X across species, such as the distinction between the avian MDA5-STING pathway and the human MAVS pathway—highlights the need to develop species-specific targeting strategies to avoid efficacy discrepancies in trans-species applications.

Collectively, DDX3X plays a pivotal role in viral infection processes. Future research should focus on deciphering the structural dynamics of DDX3X-virus interactions, the impact of host genetic backgrounds on DDX3X function, and the role of virus-host co-evolution in shaping DDX3X regulatory networks. Meanwhile, integrating artificial intelligence-based screening with high-throughput technologies to develop highly selective inhibitors and exploring combination therapies will be critical to balance antiviral efficacy with host tolerance. Ultimately, deeply unraveling the multifaceted roles of DDX3X in the virus-host interplay will provide a theoretical foundation for precision antiviral therapy and open new pathways to address emerging viral threats.

## References

[ref1] Amaya M. Brooks-Faulconer T. Lark T. Keck F. Bailey C. Raman V.. (2016). Venezuelan equine encephalitis virus non-structural protein 3 (nsP3) interacts with RNA helicases DDX1 and DDX3 in infected cells. Antivir. Res. 131, 49–60. doi: 10.1016/j.antiviral.2016.04.008, PMID: 27105836 PMC7113772

[ref2] Bei C. Zhang C. Wu H. Feng H. Zhang Y. A. Tu J. (2023). DDX3X is hijacked by snakehead Vesiculovirus phosphoprotein to facilitate virus replication via stabilization of the phosphoprotein. J. Virol. 97:e0003523. doi: 10.1128/jvi.00035-23, PMID: 36744958 PMC9972964

[ref3] Benfield C. Ren H. Lucas S. J. Bahsoun B. Smith G. L. (2013). Vaccinia virus protein K7 is a virulence factor that alters the acute immune response to infection. J. Gen. Virol. 94, 1647–1657. doi: 10.1099/vir.0.052670-0, PMID: 23580427 PMC3709632

[ref4] Brai A. Martelli F. Riva V. Garbelli A. Fazi R. Zamperini C.. (2019). DDX3X helicase inhibitors as a new strategy to fight the West Nile virus infection. J. Med. Chem. 62, 2333–2347. doi: 10.1021/acs.jmedchem.8b01403, PMID: 30721061

[ref5] Brai A. Riva V. Saladini F. Zamperini C. Trivisani C. I. Garbelli A.. (2020). DDX3X inhibitors, an effective way to overcome HIV-1 resistance targeting host proteins. Eur. J. Med. Chem. 200:112319. doi: 10.1016/j.ejmech.2020.112319, PMID: 32446036

[ref6] Brai A. Trivisani C. I. Poggialini F. Pasqualini C. Vagaggini C. Dreassi E. (2022). DEAD-box helicase DDX3X as a host target against emerging viruses: new insights for medicinal chemical approaches. J. Med. Chem. 65, 10195–10216. doi: 10.1021/acs.jmedchem.2c00755, PMID: 35899912

[ref7] Cavignac Y. Lieber D. Laib S. K. Madlung J. Lamkemeyer T. Jahn G.. (2015). The cellular proteins Grb2 and DDX3 are increased upon human cytomegalovirus infection and act in a proviral fashion. PLoS One 10:e0131614. doi: 10.1371/journal.pone.0131614, PMID: 26121620 PMC4509573

[ref8] Cheng Y. Sun Y. Wang H. Yan Y. Ding C. Sun J. (2015). Chicken STING mediates activation of the IFN gene independently of the RIG-I gene. J. Immunol. 195, 3922–3936. doi: 10.4049/jimmunol.1500638, PMID: 26392466

[ref9] Ciccosanti F. Di Rienzo M. Romagnoli A. Colavita F. Refolo G. Castilletti C.. (2021). Proteomic analysis identifies the RNA helicase DDX3X as a host target against SARS-CoV-2 infection. Antivir. Res. 190:105064. doi: 10.1016/j.antiviral.2021.105064, PMID: 33781803 PMC7997689

[ref10] Cronin S. Tejada M. A. Song R. Laval K. Cikes D. Ji M. Pseudorabies virus hijacks DDX3X, initiating an addictive "mad itch" and immune suppression, to facilitate viral spread. bio Rxiv [Preprint] (2023). doi: 10.1101/2023.05.09.539956

[ref11] De Colibus L. Stunnenberg M. Geijtenbeek T. (2022). DDX3X structural analysis: implications in the pharmacology and innate immunity. Curr. Res. Immunol. 3, 100–109. doi: 10.1016/j.crimmu.2022.05.002 35647523 PMC9133689

[ref12] Gringhuis S. I. Hertoghs N. Kaptein T. M. Zijlstra-Willems E. M. Sarrami-Forooshani R. Sprokholt J. K.. (2017). HIV-1 blocks the signaling adaptor MAVS to evade antiviral host defense after sensing of abortive HIV-1 RNA by the host helicase DDX3. Nat. Immunol. 18, 225–235. doi: 10.1038/ni.3647, PMID: 28024153

[ref13] Gu L. Fullam A. McCormack N. Hohn Y. Schroder M. (2017). DDX3 directly regulates TRAF3 ubiquitination and acts as a scaffold to co-ordinate assembly of signalling complexes downstream from MAVS. Biochem. J. 474, 571–587. doi: 10.1042/BCJ20160956, PMID: 27980081

[ref14] Guan J. Han S. Wu J. Zhang Y. Bai M. Abdullah S. W.. (2021). Ribosomal protein L13 participates in innate immune response induced by foot-and-mouth disease virus. Front. Immunol. 12:616402. doi: 10.3389/fimmu.2021.616402, PMID: 34093518 PMC8173215

[ref15] Han D. Yin W. Zhang X. Lu X. Wu N. (2023). Hsa-miR-181-5p inhibits human immunodeficiency virus type 1 replication by downregulating DDX3X expression. Virology 587:109868. doi: 10.1016/j.virol.2023.109868, PMID: 37651885

[ref16] Heaton S. M. Gorry P. R. Borg N. A. (2023). DExD/H-box helicases in HIV-1 replication and their inhibition. Trends Microbiol. 31, 393–404. doi: 10.1016/j.tim.2022.11.001, PMID: 36463019

[ref17] Jin J. Sun T. Zhang M. Cheng J. Gu J. Huang L.. (2023). EBV-encoded microRNA-BART17-3p targets DDX3X and promotes EBV infection in EBV-associated T/natural killer-cell lymphoproliferative diseases. Open Forum Infect. Dis. 10:ofad516. doi: 10.1093/ofid/ofad516, PMID: 38023563 PMC10652706

[ref18] Khadivjam B. Bonneil E. Thibault P. Lippe R. (2023). RNA helicase DDX3X modulates herpes simplex virus 1 nuclear egress. Commun. Biol. 6:134. doi: 10.1038/s42003-023-04522-w, PMID: 36725983 PMC9892522

[ref19] Ko C. Lee S. Windisch M. P. Ryu W. S. (2014). DDX3 DEAD-box RNA helicase is a host factor that restricts hepatitis B virus replication at the transcriptional level. J. Virol. 88, 13689–13698. doi: 10.1128/JVI.02035-14, PMID: 25231298 PMC4248967

[ref20] Kumar R. Singh N. Abdin M. Z. Patel A. H. Medigeshi G. R. (2017). Dengue virus capsid interacts with DDX3X-a potential mechanism for suppression of antiviral functions in dengue infection. Front. Cell. Infect. Microbiol. 7:542. doi: 10.3389/fcimb.2017.00542, PMID: 29387631 PMC5776122

[ref21] Kwon J. Choi H. Han C. (2022). A dual role of DDX3X in dsRNA-derived innate immune signaling. Front. Mol. Biosci. 9:912727. doi: 10.3389/fmolb.2022.912727, PMID: 35874614 PMC9299366

[ref22] Li N. Jiang S. Zhao J. Yang Y. Deng K. Wei L.. (2020). Molecular identification of duck DDX3X and its potential role in response to Tembusu virus. Dev. Comp. Immunol. 106:103599. doi: 10.1016/j.dci.2019.103599, PMID: 31899305

[ref23] Liu J. Huang X. Yu Y. Zhang J. Ni S. Hu Y.. (2017). Fish DDX3X exerts antiviral function against grouper nervous necrosis virus infection. Fish Shellfish Immunol. 71, 95–104. doi: 10.1016/j.fsi.2017.09.068, PMID: 28964860

[ref24] Lodola C. Secchi M. Sinigiani V. De Palma A. Rossi R. Perico D.. (2023). Interaction of SARS-CoV-2 Nucleocapsid protein and human RNA helicases DDX1 and DDX3X modulates their activities on double-stranded RNA. Int. J. Mol. Sci. 24:784. doi: 10.3390/ijms24065784, PMID: 36982856 PMC10058294

[ref25] Loureiro M. E. Zorzetto-Fernandes A. L. Radoshitzky S. Chi X. Dallari S. Marooki N.. (2018). DDX3 suppresses type I interferons and favors viral replication during arenavirus infection. PLoS Pathog. 14:e1007125. doi: 10.1371/journal.ppat.1007125, PMID: 30001425 PMC6042795

[ref26] Luo H. Liu D. Liu W. Jin J. Bi X. Zhang P.. (2023). Clinical and genetic characterization of Epstein-Barr virus-associated T/NK-cell lymphoproliferative diseases. J. Allergy Clin. Immunol. 151, 1096–1109. doi: 10.1016/j.jaci.2022.11.012, PMID: 36423698

[ref27] Mao Q. Ma S. Li S. Zhang Y. Li S. Wang W.. (2024). PRRSV hijacks DDX3X protein and induces ferroptosis to facilitate viral replication. Vet. Res. 55:103. doi: 10.1186/s13567-024-01358-y, PMID: 39155369 PMC11331664

[ref28] Mo J. Liang H. Su C. Li P. Chen J. Zhang B. (2021). DDX3X: structure, physiologic functions and cancer. Mol. Cancer 20:38. doi: 10.1186/s12943-021-01325-7, PMID: 33627125 PMC7903766

[ref29] Nelson C. Mrozowich T. Gemmill D. L. Park S. M. Patel T. R. (2021). Human DDX3X unwinds Japanese encephalitis and Zika viral 5' terminal regions. Int. J. Mol. Sci. 22:413. doi: 10.3390/ijms22010413, PMID: 33401776 PMC7795613

[ref30] Niu Q. Cheng Y. Wang H. Yan Y. Sun J. (2019). Chicken DDX3X activates IFN-beta via the chSTING-chIRF7-IFN-beta signaling axis. Front. Immunol. 10:822. doi: 10.3389/fimmu.2019.00822, PMID: 31057547 PMC6478769

[ref31] Oshiumi H. Ikeda M. Matsumoto M. Watanabe A. Takeuchi O. Akira S.. (2010). Hepatitis C virus core protein abrogates the DDX3 function that enhances IPS-1-mediated IFN-beta induction. PLoS One 5:e14258. doi: 10.1371/journal.pone.0014258, PMID: 21170385 PMC2999533

[ref32] Oshiumi H. Sakai K. Matsumoto M. Seya T. (2010). DEAD/H BOX 3 (DDX3) helicase binds the RIG-I adaptor IPS-1 to up-regulate IFN-beta-inducing potential. Eur. J. Immunol. 40, 940–948. doi: 10.1002/eji.200940203, PMID: 20127681

[ref33] Park J. T. Oh S. (2022). The translational landscape as regulated by the RNA helicase DDX3. BMB Rep. 55, 125–135. doi: 10.5483/BMBRep.2022.55.3.188, PMID: 35236544 PMC8972136

[ref34] Puhach O. Ostermann E. Krisp C. Frascaroli G. Schluter H. Brinkmann M. M.. (2020). Murine cytomegaloviruses m139 targets DDX3 to curtail interferon production and promote viral replication. PLoS Pathog. 16:e1008546. doi: 10.1371/journal.ppat.1008546, PMID: 33031466 PMC7575108

[ref35] Quaranta P. Lottini G. Chesi G. Contrafatto F. Russotto R. Macera L.. (2020). DDX3 inhibitors show antiviral activity against positive-sense single-stranded RNA viruses but not against negative-sense single-stranded RNA viruses: the coxsackie B model. Antivir. Res. 178:104750. doi: 10.1016/j.antiviral.2020.104750, PMID: 32205137

[ref36] Rao S. Lungu C. Crespo R. Steijaert T. H. Gorska A. Palstra R. J.. (2021). Selective cell death in HIV-1-infected cells by DDX3 inhibitors leads to depletion of the inducible reservoir. Nat. Commun. 12:2475. doi: 10.1038/s41467-021-22608-z 33931637 PMC8087668

[ref37] Samir P. Place D. E. Malireddi R. Kanneganti T. D. (2021). TLR and IKK complex-mediated innate immune signaling inhibits stress granule assembly. J. Immunol. 207, 115–124. doi: 10.4049/jimmunol.2100115, PMID: 34145059 PMC11631289

[ref38] Shikalov A. B. Sergeeva O. V. Zatsepin T. S. (2021). Determination of the affinity of eukaryotic DDX3 RNA helicase to the characteristic elements of mRNA secondary structure. Dokl. Biochem. Biophys. 500, 297–299. doi: 10.1134/S1607672921050173, PMID: 34697730

[ref39] Swarup A. Bolger T. A. (2024). The role of the RNA helicase DDX3X in Medulloblastoma progression. Biomolecules 14:803. doi: 10.3390/biom14070803, PMID: 39062517 PMC11274571

[ref40] Vesuna F. Akhrymuk I. Smith A. Winnard P. J. Lin S. C. Panny L.. (2022). RK-33, a small molecule inhibitor of host RNA helicase DDX3, suppresses multiple variants of SARS-CoV-2. Front. Microbiol. 13:959577. doi: 10.3389/fmicb.2022.959577, PMID: 36090095 PMC9453862

[ref41] Wang H. Ryu W. S. (2010). Hepatitis B virus polymerase blocks pattern recognition receptor signaling via interaction with DDX3: implications for immune evasion. PLoS Pathog. 6:e1000986. doi: 10.1371/journal.ppat.1000986, PMID: 20657822 PMC2904777

[ref42] Wang W. T. Tsai T. Y. Chao C. H. Lai B. Y. Wu L. Y. (2015). Y-box binding protein 1 stabilizes hepatitis C virus NS5A via phosphorylation-mediated interaction with NS5A to regulate viral propagation. J. Virol. 89, 11584–11602. doi: 10.1128/JVI.01513-15, PMID: 26355086 PMC4645663

